# Soluble CD44: quantification and molecular repartition in plasma of patients with colorectal cancer

**DOI:** 10.1038/sj.bjc.6690633

**Published:** 1999-08

**Authors:** D Masson, M G Denis, M Denis, D Blanchard, M J Loirat, E Cassagnau, P Lustenberger

**Affiliations:** Laboratoire de Biochimie Médicale, Faculté de Médecine, 44035-Nantes, France; Laboratoire de Biochimie Spécialisée, Laboratoire d'Anatomie Pathologique A, Institut de Biologie, CHU Hôtel Dieu, 44035-Nantes, France; Etablissement de Transfusion Sanguine, 44035-Nantes, France

**Keywords:** soluble CD44, colorectal cancer, plasma

## Abstract

Based on the important role of CD44 in tumour progression and metastasis, we evaluated, in a prospective study, plasma-soluble CD44 (sCD44) as a serum marker in colorectal cancer. Blood plasma specimens from 89 patients with colorectal neoplasm, 22 patients with a gastrointestinal disease and 23 healthy donors were analysed for quantitation (ELISA assay) and purification of sCD44. The concentration of sCD44, indicating the concentration of all isoforms, was significantly higher in patients with colorectal cancer and intestinal disease than in normal individuals, but no significant differences were found between the two groups. We found no association between plasma levels and staging of the colorectal cancer patients according to Astler and Coller. A two-step batch purification combining ion exchange and immunoaffinity chromatography, followed by Western blot analysis, revealed a complex pattern with a major band corresponding to the standard form of CD44 and minor bands that may correspond to larger variant forms. No particular sCD44 isoform was clearly associated with anatomopathological or biological information. © 1999 Cancer Research Campaign


British Journal of Cancer (1999) 80(12), 1995–2000
© 1999 Cancer Research Campaign
Article no. bjoc.1999.0633

### Soluble CD44: quantification and molecular repartition in plasma of patients with colorectal cancer

**D Masson[1,2], MG Denis[1,2], M Denis[2], D Blanchard[4], MJ Loirat[4], E Cassagnau[3] and P Lustenberger[1,2]**

[1]Laboratoire de Biochimie Médicale, Faculté de Médecine, 44035-Nantes, France; [2]Laboratoire de Biochimie Spécialisée et [3]Laboratoire d'Anatomie Pathologique A, Institut de Biologie, CHU Hôtel Dieu, 44035-Nantes, France; [4]Etablissement de Transfusion Sanguine, 44035-Nantes, France

**Summary** Based on the important role of CD44 in tumour progression and metastasis, we evaluated, in a prospective study, plasma-soluble CD44 (sCD44) as a serum marker in colorectal cancer. Blood plasma specimens from 89 patients with colorectal neoplasm, 22 patients with a gastrointestinal disease and 23 healthy donors were analysed for quantitation (ELISA assay) and purification of sCD44. The concentration of sCD44, indicating the concentration of all isoforms, was significantly higher in patients with colorectal cancer and intestinal disease than in normal individuals, but no significant differences were found between the two groups. We found no association between plasma levels and staging of the colorectal cancer patients according to Astler and Coller. A two-step batch purification combining ion exchange and immunoaffinity chromatography, followed by Western blot analysis, revealed a complex pattern with a major band corresponding to the standard form of CD44 and minor bands that may correspond to larger variant forms. No particular sCD44 isoform was clearly associated with anatomopathological or biological information.

**Keywords:** soluble CD44; colorectal cancer; plasma

CD44 represent a family of transmembrane glycoproteins. They are found at the cell surface in many tissues (Higashikawa et al, 1996) and in soluble form in plasma (Lucas et al, 1989). Isoforms of CD44 are attributed to alternative splicing of a single CD44 gene (Screaton et al, 1992) and post-translational modifications such as *N*- and *O*-glycosylation (Labarrière et al, 1994). Physiologically, CD44 is the main cell surface receptor of hyaluronate, an important component of the extracellular matrix (Aruffo et al, 1990). Consequently, CD44 is involved in a number of various cell–cell and cell–matrix interactions including lymphocyte migration and activation (Huet et al, 1989; Galmiche and Lustenberger, 1994; Rafi et al, 1997).

The genomic structure of CD44 reveals a striking degree of complexity and confirms the role of alternative splicing as the basis of the structural and functional diversity present in the CD44 molecules. After activation, lymphocytes transiently up-regulate CD44 isoforms expressing variant exons (Arch et al, 1992). The expression of such variants after transfection of rat CD44 cDNAs containing exons v6 and v7 confers a metastatic phenotype to non agressive rat pancreatic adenocarcinoma cell lines (Günthert et al, 1991).

The expression pattern of CD44 has been studied in various types of tissues and human malignant tumours, for instance in neuroblastoma (Combaret et al, 1996), in normal colonic mucosa and colorectal carcinomas (Gorham et al, 1996; Imazeki et al, 1996), in human digestive tract carcinomas and normal tissues (Higashikawa et al, 1996) and in ovarian cancer (Sliutz et al, 1995). These studies investigated CD44 gene expression at both RNA and protein levels. mRNA were analysed by reverse transcription polymerase chain reaction (RT-PCR), Southern blotting, or in situ hybridization. Proteins were studied by immunohistochemistry or Western blotting using specific antibodies against standard CD44 or variant isoforms. An overexpression of variant or standard CD44 has been described for most cancers (Gorham et al, 1996; Higashikawa et al, 1996), whereas a down-regulation has been associated with neuroblastoma progression (Combaret et al, 1996).

As far as colorectal cancer is concerned, the tumour cells and the cells from the normal mucosa present a similar CD44 alternative splicing pattern, but the expression level is higher in cancer cells. CD44 variants that have been associated with malignant transformation and metastasis, especially CD44v6, are commonly expressed by normal colonic epithelia, particularly the basal crypt epithelium (Gotley et al, 1996). The abnormal and elevated CD44 expression revealed by PCR is a direct product of the cancer cells (Gorham et al, 1996), but no particular CD44 transcript was clearly associated with colorectal tumour progression (Imazeki et al, 1996). Each variant exon, from v2 to v10, was detected in tumours independently of their grade. However, aberrant expression of CD44 is frequent and some isoforms might have an important role in tumour progression suggesting a complex role for CD44 in colorectal malignancy.

Colorectal cancer is the second most common cause of death from malignant disease in Western Europe and North America. There is a clear need for more sensitive serum markers. As many cell surface receptors, CD44 is present as a soluble form in extracellular fluids. sCD44 has been measured and partially characterized in human plasma (Lucas et al, 1989). In a first report, sCD44 has been proposed to be a valuable indicator of tumour growth in gastric and colorectal cancer (Guo et al, 1994). The aim of this work was to analyse, in a prospective study, the concentration and the molecular mass of sCD44 molecules in the plasma of colorectal cancer patients.

*Received 1 October 1998*
*Accepted 22 January 1999*

*Correspondence to:* P Lustenberger, Laboratoire de Biochimie Médicale, Faculté de Médecine, 1 rue G Veil, 44035 Nantes, France

### MATERIALS AND METHODS

### Blood plasma specimen

Blood samples were collected in heparinized tubes and centrifuged. Plasma was aliquoted and stored at –20°C until used. Samples from 89 patients with colorectal neoplasms were analysed for quantitation and purification of sCD44. Blood sampling was performed immediately before the beginning of surgery. Blood samples collected from 23 healthy donors and 22 patients with gastrointestinal diseases (alcoholic hepatitis, diverticulosis, Crohn's disease, sigmoiditis, gastric ulcer) were used as controls.

### Monoclonal antibodies

Monoclonal antibodies NaM10-8F4 and NaM77-9D6 were produced by BALB/c mice immunized with human erythrocytes (Blanchard et al, 1997). NaM77-9D6 was conjugated to CNBr-activated Sepharose 4B beads (Amersham Pharmacia Biotech, Uppsala, Sweden) as described by manufacturer.

### Plasma assay

Plasma levels of sCD44 were measured using the CD44std enzyme-linked immunosorbent assay (ELISA) (Bender MedSystems, Vienna, Austria). The sCD44std ELISA kit allows the quantitative detection of all circulating CD44 isoforms comprising the standard protein and the different isoforms.

CA19-9 and carcinoembryonic antigen (CEA) plasma levels were determined with commercially available microparticular enzyme immunoassay (MEIA) kits (Abbot Laboratories, Abbot Park, IL, USA) according to the manufacturer's instructions.

### Purification of CD44

The CD44 was purified from plasma using Q-Sepharose Fast Flow anion exchange chromatography (Amersham Pharmacia Biotech, Uppsala, Sweden) and NaM77-9D6-Sepharose 4B immunoaffinity. Plasma (150 ml) was diluted fivefold with buffer A (10 mM phosphate buffer, pH 6.0). The protease inhibitors 4-amidino phenylmethanesulphonyl fluoride (APMSF) and EDTA were added to a final concentration of 20 μM and 1 mM respectively. The Q-Sepharose ion exchanger column (150 ml) was equilibrated in buffer A. The diluted plasma was applied to the column at a flow rate of 2 ml $min^{-1}$. The column was then washed with 500 ml buffer A and 500 ml buffer B (10 mM phosphate buffer, 0.3 M sodium chloride, 1 mM EDTA, pH 6.0) Bound proteins were then eluted with 250 ml buffer C (10 mM phosphate buffer, 1 M sodium chloride, 1 mM EDTA, pH 6.0). The Q-Sepharose column was washed and regenerated according to Pharmacia instructions. The eluate (50 ml) was applied to NaM77-9D6-Sepharose 4B immunoaffinity column (1 ml) equilibrated in buffer C. The affinity column was washed with 10 ml buffer C at a flow rate of 1 ml $min^{-1}$. Immunoaffinity-purified CD44 was eluted from the column with 50 mM triethylamine (pH 12.0) and extensively dialysed against phosphate-buffered saline (PBS). The immunoaffinity column was then washed and regenerated. The protein concentration was determined by using the bicinchoninic acid protein assay (BCA) reagent (Pierce, Rockford, IL, USA). The sCD44 concentration was determined by using the sCD44std ELISA kit.

**Table 1** sCD44 in the plasma of healthy controls and patients

| | | sCD44 ng $ml^{-1}$ | | |
|---|---|---|---|---|
| **Patients** | ***n*** | **Mean ± s.d.** | **Range** | **Median** |
| Healthy donors | 23 | 260 ± 43 | 177–396 | 252 |
| Colorectal carcinoma | 89 | 304 ± 103 | 111–747 | 303 |
| Intestinal diseases | 22 | 337 ± 98 | 207–597 | 316 |

A batch-purification was adapted to 1-ml plasma samples. The sample was mixed with protease inhibitors APMSF and EDTA as above and was diluted with 5 ml buffer A. Q-Sepharose (1 ml) equilibrated with buffer A was added to the diluted plasma and gently mixed for 30 min at room temperature. The Q-Sepharose was then washed with buffer A (3 × 5 ml) and buffer B (2 ml). Bound proteins were eluted with buffer C (1.5 ml). The eluate was mixed for 3 h at room temperature with NaM77-9D6-Sepharose 4B (30 μl). The affinity gel was washed with buffer C (3 × 0.15 ml) and immunoaffinity-purified CD44 was eluted with 0.25 ml triethylamine 50 mM, pH 12.0.

### Western blot analysis

Proteins were precipitated with trichloracetic acid (20% w/v) overnight at 4°C. After centrifugation and washing with –20°C acetone, proteins were dissolved in Laemmli's sample buffer without reducing reagent. To determine the size of soluble forms of CD44 in plasma, the proteins were separated by gradient (4–10%) sodium dodecyl sulphate polyacrylamide gel electrophoresis (SDS-PAGE) and detected by immunoblotting. Proteins were transferred to nitrocellulose membranes. Filters were saturated for 1 h with non-fat dry milk (3% w/v) in Tris-buffered saline (TBS) and subsequently incubated with NaM10-8F4 (diluted 1/200) in TBS supplemented with 1% non-fat dry milk for 1 h under continuous stirring at room temperature. After exhaustive rinsing with TBS containing 0.5% Tween-20, membranes were incubated with alkaline phosphatase-conjugated goat anti-mouse IgG (Sigma, St Louis, MO, USA) in TBS supplemented with 1% non-fat dry milk for 1 h. After washing with TBS containing 0.5% Tween-20, the protein bands were detected using BCIP/NBT system (Nitro blue tetrazolium/5-bromo-4-chloro-3-indolyl phosphate) (Promega, Madison, WI, USA). Prestained molecular weight markers (Sigma) were used as standards.

### RESULTS

### Plasma sCD44 measurement

Blood samples were collected before the patients underwent surgical therapy. The mean age of these patients was 66.3 ± 12.7 years (range 32–93) with 42 women and 47 men. The results of sCD44 plasma levels (means, medians and standard deviations) are presented in Table 1.

The measured mean level of healthy donors was 260 ± 43 ng $ml^{-1}$. sCD44 plasma levels in patients with colorectal carcinoma or intestinal diseases were statistically significantly higher than in healthy controls ($P$ = 0.001). In contrast, we found no significant difference between sCD44 level in patients with a colorectal cancer or a gastrointestinal disease.

© 1999 Cancer Research Campaign

**Table 2** sCD44 and clinicobiological parameters of 89 colorectal cancer patients

| | | sCD44 ng ml$^{-1}$ | | |
|---|---|---|---|---|
| **Parameter** | **Patients** | **Mean ± s.d.** | **Range** | **Median** |
| Sex | | | | |
| Female | 42 | 303 ± 90 | 126–501 | 304 |
| Male | 47 | 305 ± 103 | 111–747 | 303 |
| Age (years) | | | | |
| ≤ 65 | 39 | 297 ± 91 | 111–513 | 303 |
| ≥ 65 | 50 | 304 ± 103 | 111–747 | 303 |
| Tumour location | | | | |
| Colon | 48 | 318 ± 107 | 123–747 | 318 |
| Rectum+sigmoid | 34 | 290 ± 100 | 111–513 | 290 |
| Unknown | 7 | | | |
| Astler–Coller's stage | | | | |
| A | 6 | 351 ± 82 | 213–462 | 361 |
| B1 | 13 | 273 ± 82 | 129–450 | 255 |
| B2 | 26 | 290 ± 102 | 123–513 | 285 |
| C1 | 2 | 290 ± 51 | 219–321 | 270 |
| C2 | 30 | 312 ± 121 | 111–747 | 303 |
| D | 12 | 336 ± 66 | 252–513 | 327 |
| CEA (ng ml$^{-1}$) | | | | |
| ≤ 5 | 53 | 305 ± 101 | 126–513 | 294 |
| ≥ 5 | 36 | 305 ± 106 | 111–747 | 303 |
| CA 19.9 (U ml$^{-1}$) | | | | |
| ≤ 37 | 67 | 295 ± 94 | 123–513 | 294 |
| ≥ 37 | 22 | 305 ± 122 | 111–747 | 318 |

The clinical staging of these patients was performed according to Astler and Coller (1954). Plasma concentrations of tumour markers CEA and CA19.9 in these samples were also determined. Plasma levels of sCD44, grouped by age, sex, tumour location, Astler–Coller's stage, CEA and CA19.9 plasma levels are shown in Table 2. No statistically significant differences were found between sCD44 plasma levels when samples were grouped according Astler–Coller's stage. The sCD44 concentration was not correlated with the CEA or CA19.9 serum levels, using the cut-off values of 5 ng ml$^{-1}$ and 37 U ml$^{-1}$ respectively.

### Purification of sCD44

The concentration of sCD44, indicating the concentration of all isoforms, was significantly higher in colorectal cancer than in normal individuals, but no association was found between sCD44 and clinical parameters. One possible explanation is that only some isoforms might play an important role in tumour progression. To investigate if one isoform was more specifically involved, isoform-specific ELISA can be used. Alternatively, immunoprecipitation and Western blot analysis can lead to the simultaneous identification of several isoforms. Since no particular CD44 variant exon was clearly associated with colorectal tumour progression, we decided to analyse the molecular mass of sCD44 molecules in plasma of colorectal cancer patients by immunopurification and Western blot analysis.

We first immunoprecipitated sCD44 from plasma using NaM77-9D6 anti-CD44 antibodies covalently linked to agarose beads. The immunoprecipitated proteins were then separated by gradient SDS-PAGE and revealed with NaM10-8F4 anti-CD44 antibodies, followed by anti-mouse immunoglobulins. As shown

**Table 3** Purification of soluble CD44

| **Step** | **Volume (ml)** | **sCD44 (ng ml$^{-1}$)** | **Proteins (g)** | **sCD44 (μg)** | **Purification (-fold)** | **Yield (%)** |
|---|---|---|---|---|---|---|
| Plasma | 150 | 429 | 10 | 64.3 | 1 | 100 |
| NaM77-9D6-Sepharose eluate | 4 | 3000 | $3.04\ 10^{-4}$ | 12 | 6139 | 19 |

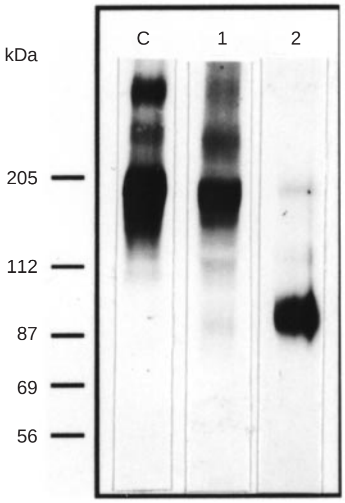

**Figure 1** Immunopurified sCD44 obtained from plasma of a healthy donor by NaM77-9D6-Sepharose 4B immunoaffinity chromatography. Staining with irrelevant antibody as negative control (lane C). Revelation with NaM10-8F4 antibody (lane 1). Lysate of peripheral blood lymphocytes (5 × 10$^5$ cells) used as positive control for CD44std (lane 2)

in Figure 1 (lane 1), a strong signal was seen. However, when an irrelevant monoclonal antibody was used for the detection, a similar pattern was obtained (Figure 1, lane C), indicating that these proteins bound non-specifically to the immunoaffinity matrix. No such bands were detected when proteins extracted from lymphocytes were used as a positive control (Figure 1, lane 2), indicating that the contaminating proteins originated from plasma.

We therefore included as a first step an anion-exchange chromatography. Most of the immunoglobulins and other plasma proteins which bound non-specifically to NaM77-9D6-Sepharose were removed when the column was washed with 0.3 M sodium chloride (Figure 2A, lane 1). The final 1 M sodium chloride elution was found to contain the CD44 molecules, which were further purified by immunoaffinity chromatography (Figure 2A, lane 2). This two-step purification procedure resulted in an ~6000-fold purification of sCD44 with a final yield of 19% (Table 3). Western blot analysis of sCD44 purified from the plasma of a healthy donor revealed the presence of a prominent band with a molecular weight of 85–95 kDa and larger proteins with molecular weight of 100, 140, 180 and 240 kDa (Figure 2A, lane 2). The most prominent species of sCD44 and the standard CD44 expressed by lymphocytes displayed a similar migration (Figure 2A, lane 3). The additional immunostained bands of soluble CD44 are larger forms that may correspond to CD44v and/or to different levels of glycosylation. Duplicate lane 2, stained with an irrelevant antibody as a negative control, revealed no non-specific band (Figure 2A, lane C).

© 1999 Cancer Research Campaign 

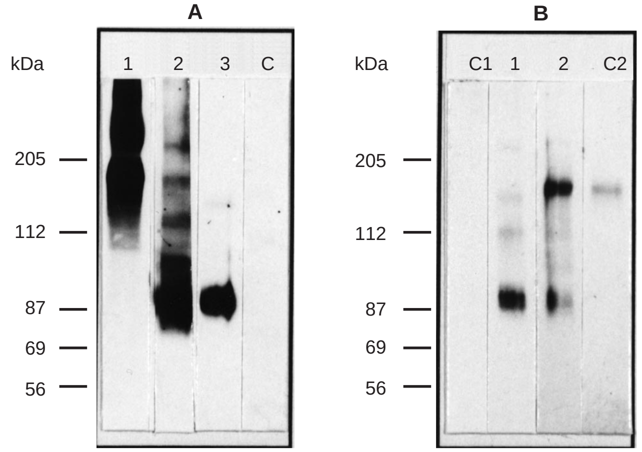

**Figure 2** Western blot analysis of purified sCD44. (**A**) Non-specific proteins removed by first ion exchange chomatographic step (lane 1). Eluate obtained from plasma of a healthy donor by two-step column purification (lane 2). Lysate of peripheral blood lymphocytes ($5 \times 10^5$ cells) used as control for CD44std (lane 3). Duplicate lane 2 stained with irrelevant antibody as negative control (lane C) (**B**) Eluate (25 μl) obtained from plasma of a healthy donor by two-step column purification (lane 1) and eluate from batch purification of 1 ml specimen of the same serum (lane 2). Duplicates lane 1 and lane 2 stained with irrelevant antibody as negative control (respectively lane C1 and C2)

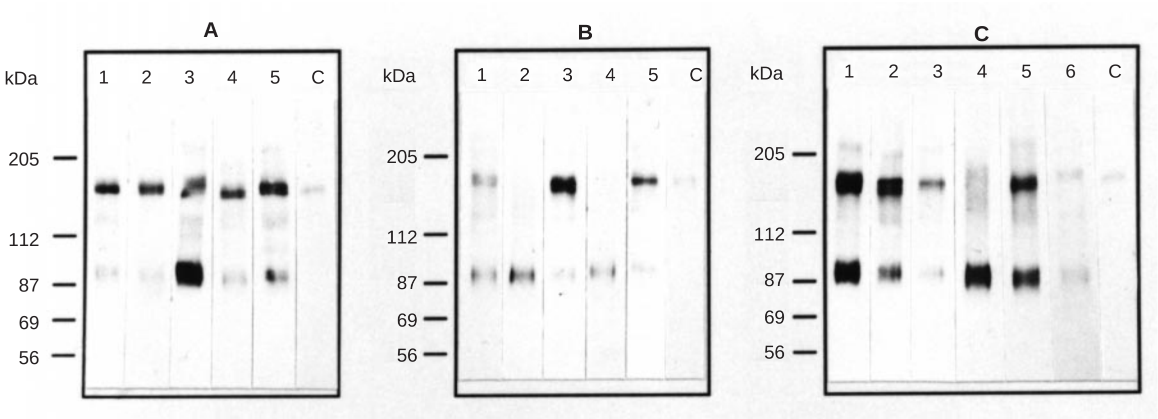

**Figure 3** Western blot analysis of sCD44 from patients. (**A**) Five healthy donors, sCD44 concentrations are respectively (1) 237 ng $ml^{-1}$, (2) 246 ng $ml^{-1}$, (3) 300 ng $ml^{-1}$, (4) 294 ng $ml^{-1}$, (5) 396 ng $ml^{-1}$; (**B**) five colorectal cancer Astler–Coller's stage B patients, sCD44 concentrations are respectively (1) 285 ng $ml^{-1}$, (2) 366 ng $ml^{-1}$, (3) 123 ng $ml^{-1}$, (4) 303 ng $ml^{-1}$, (5) 129 ng $ml^{-1}$; (**C**) six colorectal cancer Astler–Coller's stage C patients, sCD44 concentrations are respectively (1) 321 ng $ml^{-1}$, (2) 456 ng $ml^{-1}$, (3) 747 ng $ml^{-1}$, (4) 309 ng $ml^{-1}$, (5) 186 ng $ml^{-1}$, (6) 288 ng $ml^{-1}$. For each group, a control (plasma pool) stained with irrelevant antibody as negative control (lane C)

We then scaled down this purification and set up a batch-purification of sCD44 from 1-ml plasma sample. As illustrated in Figure 2B, sCD44 purified by column chromatography or batch-wise presented similar pattern. Duplicate lane 2, stained with irrelevant antibody as a negative control revealed a faint non-specific band corresponding to a molecular weight of 150 kDa (Figure 2B, lane C2). When several samples of the same plasma were subjected to purification, identical patterns were obtained, indicating that this procedure is reproducible (data not shown).

sCD44 was purified from 21 colorectal carcinoma patients, ten intestinal disease patients and six healthy donors. sCD44 size heterogeneity was observed. All the previous described immunostained bands were revealed but with great differences in intensity. The patterns obtained for five healthy donors (Figure 3A), five stage B patients according to Astler–Coller (Figure 3B) and six stage C patients (Figure 3C) are presented. There is clearly a great heterogeneity, both in size and intensity of the bands, even for a homogenous group of patients. This diversity is also important between healthy donors and disease patients. No immunostained band seemed to be associated with the presence of tumour. There is no clear difference between the colorectal carcinoma Astler–Coller's stage B patients group and the colorectal carcinoma Astler–Coller's stage C patients group, although the heterogeneity and the intensity of the bands were greater in the Astler–Coller's stage C patients group.

### DISCUSSION

Based on the important biological role of CD44 in tumour progression, several studies have suggested the potential use of soluble

 *© 1999 Cancer Research Campaign*

CD44 as a marker of disease progression (Guo et al, 1994; De Rossi et al, 1997; Ristamäki et al, 1998). For instance in ovarian cancer, the concentration of sCD44std was found to be higher and sCD44v5 to be lower than in healthy controls (Zeimer et al, 1997). A lower concentration of CD44v5 has also been reported for prostate cancer and renal cancer (Jung et al, 1996; Lein et al, 1996). In the case of renal cancer, another report (Kan et al, 1996) showed that the concentrations of sCD44std and sCD44 splice isoforms sharing exon v6 were significantly higher than in normal individuals, but there was no correlation between these concentrations and clinico-pathological parameters. In breast cancer, plasma sCD44v5 and v6 were significantly elevated in patients with node-positive breast cancer (Martin et al, 1997). In gastric carcinoma, the concentrations of sCD44v5 and v6 were elevated in gastric carcinoma patients. Moreover, plasma sCD44v5 concentrations correlated with tumour burden, lymph node involvement and the presence of metastasis (Harn et al, 1996).

In a first report, Guo et al (1994), using a home-made ELISA which detects CD44std as well as isoforms, showed that plasma sCD44 concentration was elevated in advanced colon cancer and correlated with tumour burden and metastasis. During completion of our manuscript, a second study demonstrated that both sCD44std and sCD44v6 levels were significantly higher in colorectal cancer and chronic inflammatory bowel disease than in normal individuals (Weg-Remers et al, 1998). The concentrations of sCD44 we determined for colorectal cancer patients are in agreement with the values reported by other groups using the same ELISA kit. Our data show that a significant elevation of plasma concentrations of sCD44 is found in patients with colorectal cancer or chronic inflammatory bowel disease when compared to healthy controls. There is no difference between the two groups of patients. The sCD44 level is not correlated with the presence of tumour and does not reflect the neoplastic progression or the metastatic state.

In order to investigate whether isoforms of sCD44 were associated with tumour progression or not, we designed an efficient purification procedure which allowed us to analyse simultaneously a large number of these variant molecules. This purification can be distinguished from the previously reported techniques by the quality of the sCD44 isoforms revealed by immunoblotting. A major point lies in the use of an anion exchange chromatography, which allowed us to get rid of contaminant proteins, although the batch procedure was not as efficient as the column purification. The main problem encountered in the purification of CD44 is related to the anionic nature of the protein. In the immunoaffinity step, the binding of sCD44 onto the immobilized monoclonal antibody is not only based on antigen–antibody recognition, but also on strong ionic interactions between anionic (CD44) and cationic (Ig) proteins. This led us to use triethylamine 50 mM, pH 12.0 for the elution step. These conditions preclude the obtention of great amount of CD44, explaining the somehow limited yield. Finally, both NaM77-9D6 and NaM10-8F4 epitopes are located on the constant part of CD44 within the extracellular domain of all CD44 isoforms. Therefore, all isoforms are co-purified and revealed on the immunoblot.

Purification followed by Western blot analysis revealed a complex pattern. A major band with molecular weight of 85–95 kDa corresponding to the standard form of CD44 was always seen. Minor bands, with molecular weight of 100, 140, 180 and 240 kDa, were not consistently present in the plasma of healthy donors and colorectal cancer patients. No particular CD44 band seemed to be associated with colorectal tumour progression. The sCD44 pattern is not correlated with the presence of tumour and does not reflect the neoplastic progression or the metastatic state.

The cellular origin of sCD44 isoforms in human plasma is still unknown. The soluble forms might derive from proteolytic release of extracellular domain of membranous CD44 or by alternative splicing. Studies of cell culture medium of lymphocytes or tumour cells have indicated that sCD44 can be generated, at least in part, by proteolytic cleavage of membrane-bound CD44 (Ristamäki et al, 1997), and metalloprotease and serine protease activities have been shown to be involved in this process (Bazil and Stominger, 1994). In mouse a stop codon within a new exon (v10) provides a molecular basis for alternative synthesis of sCD44 isoforms (Yu and Toole, 1996). Synthesis of sCD44 via alternative splicing provides a mechanism of production that is subject to rigourous cellular control and thus may be important in the regulation of CD44 activity. However, shedding from lymphocytes seems to represent one major origin of the soluble CD44 isolated from plasma, generating molecules with biological functions comparable to that of the membrane isoforms (Ristamäki et al, 1997).

Cleavage of membrane molecules may represent a mechanism producing soluble forms that have various regulatory functions and this process is markedly influenced by the activity of the immune system (Katoh et al, 1994). So the predominant origin of sCD44 variants in human plasma might be related to this activity. It has been proposed that sCD44 may act as an antagonist of cellular CD44, especially in tumorigenesis and immune responses. In fact, soluble adhesion molecules might act as antiadhesive agents. Shedding of CD44 occurs during immune activation, in inflammatory conditions and in neoplasia. This might explain why sCD44 levels are elevated in our group of gastrointestinal diseases patients as well as in the cancer patients group.

Two main processes are responsible for the complex pattern of sCD44 seen after purification: the alternative splicing of nine exons; and post-translational modifications such as *N*- and *O*-linked glycosylation, addition of chondroitin, or heparan sulphate side-chains. In order to determine whether sCD44 isoforms can be of value in colorectal cancer survey, we will now attempt to identify these different plasma isoforms and characterize their glycosylation status.

### ACKNOWLEDGEMENTS

We wish to thank Prof. LeBorgne and Prof. LeHur (Clinique Chirurgicale II, Hôtel-Dieu, Nantes, France), Prof. LeNeel and Dr LeTessier (Clinique Chirurgicale A, Hôtel-Dieu, Nantes, France) and Prof. Galmiche (Gastroentérologie, Hôtel-Dieu, Nantes, France) for providing us with the blood specimen. Mrs MN Hervé is gratefully acknowledged for helpful technical assistance.

### REFERENCES

Arch R, Wirth K, Hofmann M, Ponta H, Matzku S, Herrlich P and Zöller M (1992) Participation in normal immune responses of a metastasis-inducing splice variant of CD44. *Science* **257**: 682–685

Astler VB and Coller FA (1954) The prognostic significance of direct extension of carcinoma of the colon and rectum. *Ann Surg* **139**: 846–851

Aruffo A, Stamenkovic I, Melnick M, Underhill CB and Seed B (1990) CD44 is the principal cell surface receptor for hyaluronate. *Cell* **61**: 1303–1313

Bazil V and Strominger JL (1994) Metalloprotease and serine protease are involved in cleavage of CD43, CD44, and CD16 from stimulated human granulocytes. *J Immunol* **152**: 1314–1322

© 1999 Cancer Research Campaign 

Blanchard D, Petit-LeRoux Y, Willem C and Loirat MJ (1997) Flow cytometry and immunoblotting analysis of monoclonal antibodies directed to CD-related antigens. *Transfus Clin Biol* **4**: 153–156

Combaret V, Gross N, Lasset C, Frappaz D, Peruisseau G, Philip T, Beck D and Favrot MC (1996) Clinical relevance of CD44 cell-surface expression and N-*myc* gene amplification in a multicentric analysis of 121 pediatric neuroblastomas. *J Clin Oncol* **14**: 25–34

De Rossi G, Marroni P, Paganuzzi M, Mauro FR, Tenca C, Zarcone D, Velardi A, Molica A and Grossi CE (1997) Increased serum levels of soluble CD44 standard, but not of variant isoforms v5 and v6, in B cell lymphocytic leukemia. *Leukemia* **11**: 134–141

Galmiche A and Lustenberger P (1994) CD44, invasion tumorale et métastase. *Med Sci* **10**: 1282–1291

Gorham H, Sugino T, Woodman AC and Tarin D (1996) Cellular distribution of CD44 gene transcripts in colorectal carcinomas and in normal colonic mucosa. *J Clin Pathol* **49**: 482–488

Gotley DC, Fawcett J, Walsh MD, Reeder JA, Simmons DL and Antalis TM (1996) Alternatively spliced variants of the cell adhesion molecule CD44 and tumour progression in colorectal cancer. *Br J Cancer* **74**: 342–351

Günthert U, Hofmann M, Rudy W, Reber S, Zöller M, Haußmann I, Matzku S, Wenzel A, Ponta H and Herrlich P (1991) A new variant of glycoprotein CD44 confers metastatic potential to rat carcinoma cells. *Cell* **65**: 13–24

Guo YJ, Liu G, Wang X, Jin D, Wu M, Ma J and Sy MS (1994) Potential use of soluble CD44 in serum as indicator of tumor burden and metastasis in patients with gastric or colon cancer. *Cancer Res* **54**: 422–426

Harn HJ, Ho LI, Shyu RY, Yuan JS, Lin FG, Young TH, Liu CA, Tang HS and Lee WH (1996) Soluble CD44 isoforms in serum as potential markers of metastatic gastric carcinoma. *J Clin Gastroenterol* **22**: 107–110

Higashikawa K, Yokozaki H, Ue T, Taniyama K, Ishikawa T, Tarin D and Tahara E (1996) Evaluation of CD44 transcription variants in human digestive tract carcinomas and normal tissues. *Int J Cancer* **66**: 11–17

Huet S, Groux H, Caillou B, Valentin H, Prieur AM and Bernard A (1989) CD44 contributes to T cell activation. *J Immunol* **143**: 798–801

Imazeki F, Yokosuka O, Yamaguchi T, Ohto M, Isono K and Omata M (1996) Expression of variant CD44-messenger RNA in colorectal adenocarcinomas and adenomatous polyps in humans. *Gastroenterology* **110**: 362–368

Kan M, Kanayama HO, Naruo S, Tsuji M, Kojima K, Kurokawa Y and Kagawa S (1996) Serological evaluation of soluble CD44 in renal cancer. *Jpn J Cancer Res* **87**: 1191–1194

Katoh S, McCarthy JB and Kincade PW (1994) Characterization of soluble CD44 in the circulation of mice. *J Immunol* **153**: 3440–3449

Labarriere N, Piau JP, Otry C, Denis M, Lustenberger P, Meflah K and Le Pendu J (1994) Level of H-blood group expression in CD44v modulates lak cell lysis sensitivity and tumorigenicity of rat colon carcinoma cells. *Cancer Res* **54**: 6275–6281

Lein M, Weiß S, Jung K, Schnorr D and Loening S (1996) Soluble CD44 variants in serum of patients with prostate cancer and other urological malignancies. *Prostate* **29**: 334–335

Lucas MG, Green AM and Telen MJ (1989) Characterization of serum *In* (*Lu*)-related antigen: identification of a serum protein related to erythrocyte p80. *Blood* **73**: 596–600

Martin S, Jansen F, Bokelmann J and Kolb H (1997) Soluble CD44 splice variants in metastasizing human breast cancer. *Int J Cancer (Pred Oncol)* **74**: 443–445

Rafi A, Nagarkatti M and Nagarkatti PS (1997) Hyaluronate-CD44 interactions can induce murine B-cell activation. *Blood* **89**: 2901–2908

Ristamäki R, Joensuu H, Hagberg H, Kalkner KM and Jalkanen S (1998) Clinical significance of circulating CD44 in non-Hodgkin's lymphoma. *Int J Cancer (Pred Oncol.)* **79**: 221–225

Ristamäki R, Joensuu H, Grön-Virta K, Salmi M and Jalkanen S (1997) Origin and function of circulating CD44 in non-Hodgkin's lymphoma. *J Immunol* **158**: 3000–3008

Screaton GR, Bell MV, Jackson DG, Cornelis FB, Geth U and Bell JI (1992) Genomic structure of DNA encoding the lymphocyte homing receptor CD44 reveals at least 12 alternatively spliced exons. *Proc Natl Acad Sci USA* **89**: 12160–12164

Sliutz G, Tempfer C, Winkler S, Kohlberger P, Reinthaller A and Kainz C (1995) Immunohistochemical and serological evaluation of CD44 splice variants in human ovarian cancer. *Br J Cancer* **72**: 1494–1497

Weg-Remers S, Hildebrandt U, Feifel G, Moser C, Zeitz M and Stallmach A (1998) Soluble CD44 and Cd44v6 serum levels in patients with colorectal cancer are independent of tumor stage and tissue expression of CD44v6. *Am J Gastroenterol* **93**: 790–794

Yu Q and Toole BP (1996) A new alternatively spliced exon between v9 and v10 provides a molecular basis for synthesis of soluble CD44. *J Biol Chem* **271**: 20603–20607

Zeimer AG, Widschwendter M, Uhl-Steidl M, Muller-Holzner E, Daxenbichler G, Marth C and Dapunt O (1997) High serum levels of soluble CD44 variant isoform v5 are associated with favourable clinical outcome in ovarian cancer. *Br J Cancer* **76**: 1646–1651

*© 1999 Cancer Research Campaign*
